# Gastrointestinal pathology in Good’s syndrome, thinking beyond common variable immunodeficiency: a clinicopathological observation

**DOI:** 10.1007/s00428-025-04069-7

**Published:** 2025-03-26

**Authors:** Pooja Navale, Ofer Zimmerman, James Wedner, Anupam Pande, Samuel Ballentine

**Affiliations:** 1 Department of Pathology, Cooper University Health Care, 1 Cooper Plaza, Pavilion Building P065, Camden, NJ 08103 USA; 2 Department of Medicine, Washington University in St Louis, St Louis, MO USA; 3 Department of Pathology, Washington University in St Louis, St Louis, MO USA

**Keywords:** Immunodeficiency, Gastrointestinal biopsy, Good’s syndrome, CVID

## Abstract

Good’s syndrome (GS) is a rare immunodeficiency associated with thymoma, characterized by increased susceptibility to bacterial, viral, and fungal infections, along with autoimmune manifestations. Gastrointestinal symptoms are common in GS, yet its clinical and histopathological features remain underrecognized. Due to significant overlap in clinical presentation and immunological profiles, GS is frequently misdiagnosed as common variable immunodeficiency (CVID). While gastrointestinal pathology in CVID has been well-documented, data on gastrointestinal manifestations in GS are limited. In this study, we analyzed two cases of GS, both demonstrating marked reduction of plasma cells across multiple gastrointestinal sites, with variable intraepithelial lymphocytosis and mild to moderate villous blunting in the duodenum and features secondary to chronic norovirus infection in one case, and an invasive adenocarcinoma with notable depletion of lamina propria plasma cells in the other. Accurate recognition of GS is essential for pathologists and clinicians, given its poorer prognosis compared to CVID.

## Introduction

Good’s syndrome (GS) is an acquired immunodeficiency disorder associated with thymoma and characterized by low or absent B cells, hypogammaglobulinemia, and defects in cell-mediated immunity increasing their susceptibility to recurrent bacterial, viral, and fungal infections. Dr. Robert Alan Good first described an uncanny association between thymoma and adult-onset immunodeficiency [1]. The precise definition of GS remains vague and relies on the inclusion of thymic epithelial neoplasm and immunodeficiency, with a higher propensity for infections and autoimmunity [2].

GS is likely an underappreciated form of immunodeficiency and is often mistaken for common variable immunodeficiency (CVID) due to their overlapping clinical and immunological features [3]. Although patients with GS present with gastrointestinal symptoms like diarrhea [3], there is sparse literature on histomorphological spectrum in gastrointestinal tract of GS patients. We reviewed clinical records and histopathologic material in two cases of GS patients in an attempt to study the morphological features and immunologic milieu within their pathology specimens.

## Material and methods

Patient data was collected under Wash U IRB. Patients with Good’s syndrome were identified from the records of the Allergy and Immunology Division at Washington University School of Medicine in St. Louis. The surgical pathology files were then queried for available gastrointestinal specimens (biopsy or resection). Of the four identified Good’s syndrome patients, gastrointestinal surgical pathology specimens were available for two patients. Material available for review included biopsies (× 9) and resection specimens (× 2). H&E-stained slides were available for all the above.

Electronic medical records were available for these patients, and pertinent clinical information was recorded.

## Results

Case no. 1. A 67-year-old male with a history of thymectomy was referred to immunology after having found to have severe hypogammaglobulinemia (IgG at 287 mg/dL, IgA at 19 mg/dL with undetectable IgM and IgE). CD3^+^ level was 1100 (622–2402 cells/uL), CD4^+^ level was 247 (359–1519 cells/uL), CD8^+^ level was normal at 543 (187–781 cell/uL), and NK cell level was mildly decreased at 74 (6–467uL), during an immune workup for recurrent pulmonary infections and chronic diarrhea few years later. He was initiated on intravenous immunoglobulin infusions (IVIg). Around the same time, he was diagnosed with norovirus infection. Workup for other autoimmune disorders such as autoimmune enteritis, autoimmune hepatitis, and celiac disease was negative. Due to continued abdominal pain and cramping, patient underwent push enteroscopy. The patient continued to complain of diarrhea and protein-losing enteropathy with > 100 lb weight loss and was started on total parental nutrition. He was also diagnosed with acute necrotizing cholecystitis and underwent cholecystectomy.

**Fig. 1:**
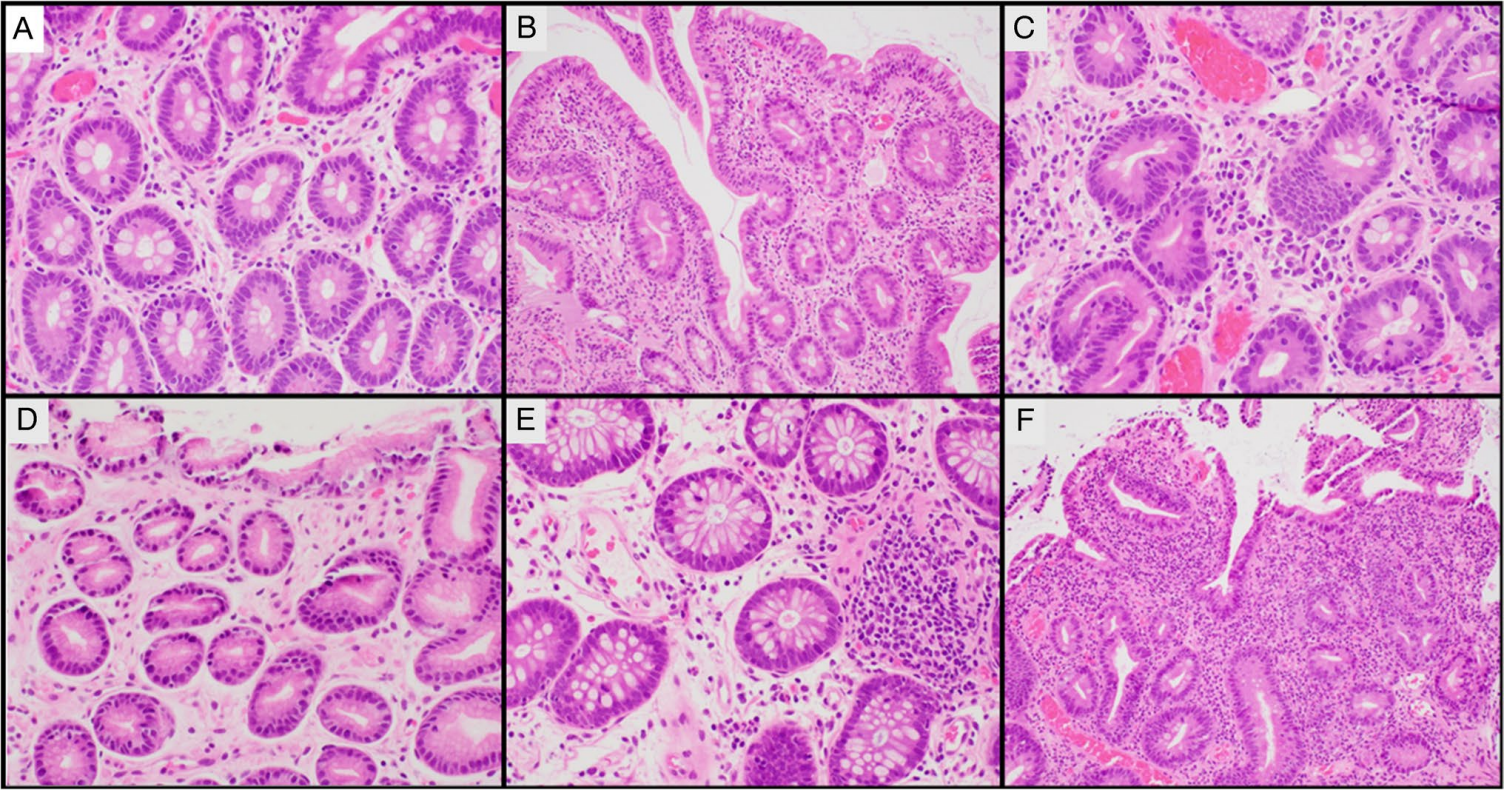
Case no. 1. **A** Duodenal biopsies showing markedly depleted lamina propria, with only scattered lymphocytic inflammation and rare plasma cells. **B** Duodenal mucosa demonstrating patchy areas of increased intraepithelial lymphocytosis and villous atrophy. **C** Duodenal mucosa in some areas show rare foci of plasma cells aggregates. **D** Stomach antral mucosa with no plasma cell in the lamina propria. **E** Colonic mucosa showing markedly depleted lamina propria inflammation. **F** Ileal mucosa with active neutrophilic enteritis

**Fig. 2:**
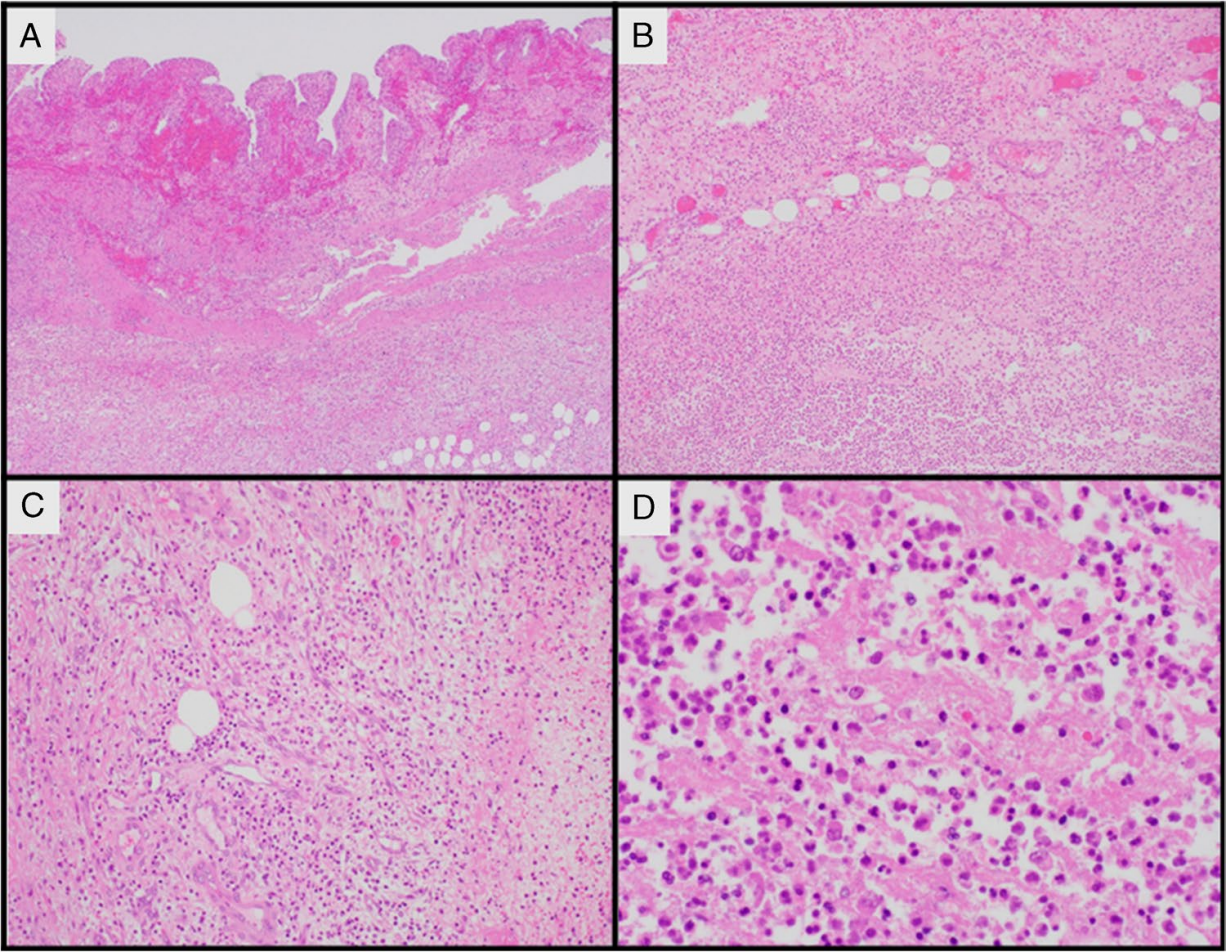
Case no. 1. Phlegmonous cholecystitis. **A**, **B** Sections from the gall bladder wall show marked acute inflammation with edema. **C**, **D** Higher power views of the inflammatory infiltrate demonstrate predominant acute neutrophilic inflammation without significant lymphoplasmacytic component

Microscopic description: Biopsies from the duodenum and jejunum revealed a noticeable decrease in lamina propria inflammatory infiltrate, with markedly reduced plasma cell infiltrate (Fig. 1A). Duodenum showed variable intraepithelial lymphocytosis (focally up to 120 intraepithelial lymphocytes [IELs] per 100 enterocytes) and mild to moderate villous blunting (Fig. 1B). Rare aggregates of plasma cells were identified (Fig. 1C). Stomach biopsies demonstrated no plasma cells (Fig. 1D). Colon biopsies were largely unremarkable except for marked lamina propria depletion, with rare plasma cells and lymphocytes (Fig. 1E). Biopsies from the terminal ileum showed acute neutrophilic ileitis (Fig. 1F). Rare plasma cells were noted in the depleted lamina propria, with no noticeable intraepithelial lymphocytes, eosinophils, macrophages, granulomatous inflammation, lymphoid hyperplasia, or organisms. Sections from the cholecystectomy demonstrated acute phlegmonous and gangrenous cholecystitis characterized by marked mucosal ulceration, transmural wall edema, and stromal reactive changes (Fig. 2A–B). The inflammatory infiltrates are essentially devoid of chronic inflammatory cells, including absence of plasma cell component (Fig. 2C–D). No granulomas, organisms, macrophages, or lymphoid hyperplasia.

Case no. 2. A 59-year-old male with a history of COPD/asthma had presented for workup of recurrent pneumonias, adenovirus, and sepsis requiring frequent hospitalizations, 6 years after thymectomy. Evaluation found significant low serum IgG at 261 mg/dL, IgA at 23 mg/dL, and IgM at 17 mg/dL. IgG subclasses were all low. Lymphocyte proliferation panel showed normal lymphocyte proliferation response to PHA. Lymphocyte subpopulation showed normal percentages in all populations. He was started on monthly 35 g IVIg infusions but continued to have episodes of pneumonia requiring antibiotics. The patient was diagnosed with locally advanced rectal cancer later and underwent neoadjuvant FOLFOX chemoradiation therapy; however, the chemotherapy was put on hold due to frequent infections requiring hospitalization. Subsequently, a resection was performed.

**Fig. 3:**
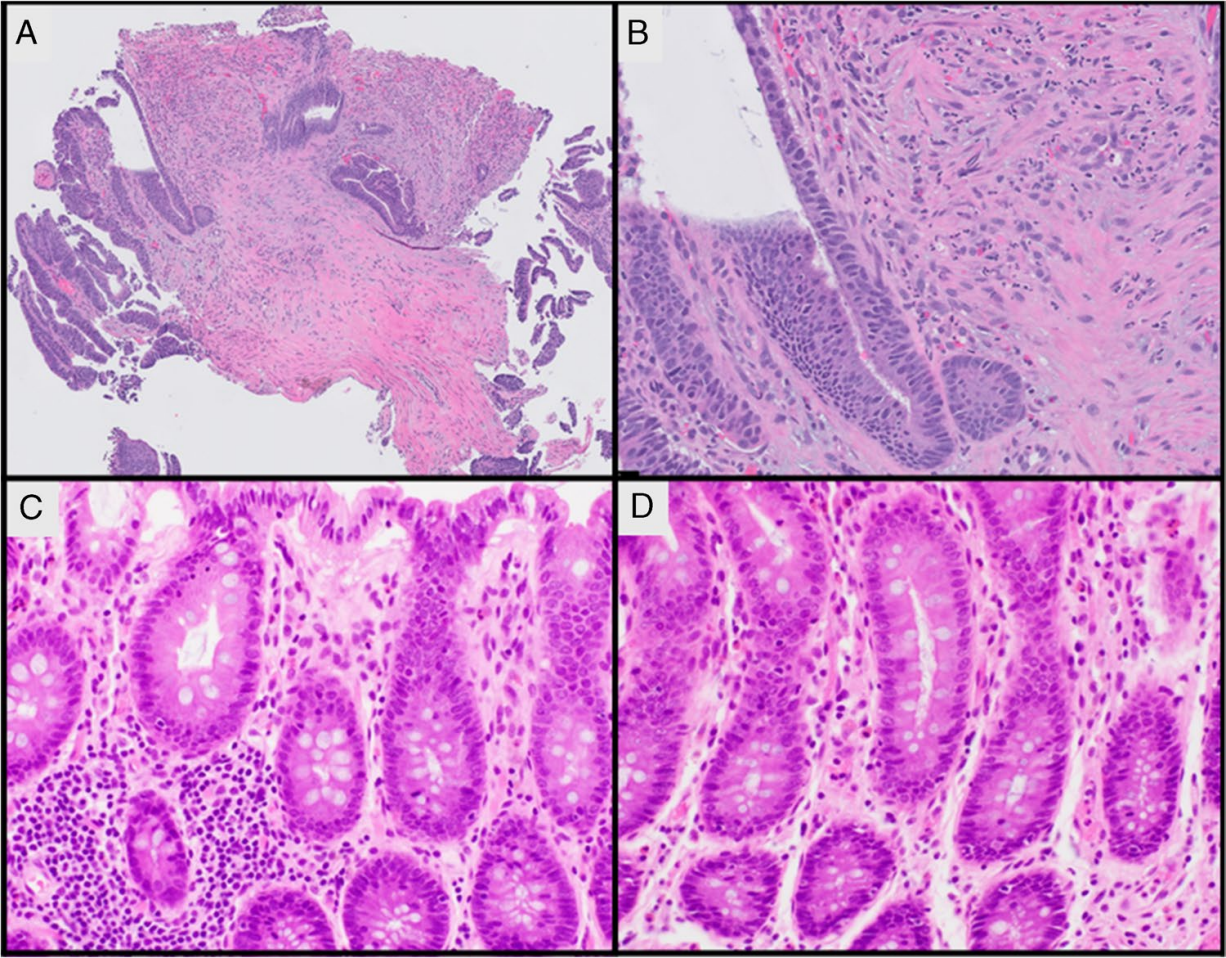
Case no. 2. **A**, **B** Rectal mass biopsies show invasive adenocarcinoma associated with desmoplastic stroma and acute inflammation. **C**, **D** Background colonic mucosa from (resection specimen) shows lamina propria devoid of plasma cells

Microscopic description: Rectal mass biopsies and resection specimen showed invasive moderately differentiated adenocarcinoma. Ulceration and inflammation were noted, composed mostly of neutrophilic infiltrates and rare eosinophils. Plasma cells were not visible. The background non-tumorous colonic mucosa was also significant for lack of lamina propria plasma cells. No increase in apoptosis, granulomas, extensive foamy histiocytes, or follicular lymphoid hyperplasia was noted elsewhere in the colon (Fig. 3).

## Discussion

Good’s syndrome (GS) is a unique form of acquired immunodeficiency. Despite the acquired nature of the disease and likely adult onset (median age at diagnosis was 58 years) [2], GS may rarely present in children [4]. A diagnosis of GS should be considered in an older individual with a medical history of thymoma with recurrent infectious complications. GS patients commonly present with gastrointestinal complaints secondary to infections and malabsorption, with approximately 50% patients with diarrheal symptoms [3]. Despite the common GI manifestations, data on the gastrointestinal histopathology in GS is sparse.

Our cases showed a common theme of markedly decreased plasma cells in the lamina propria, with slight variations, in line with the previous reports [3]. Additionally, duodenal biopsies from case no. 1 show focal intraepithelial lymphocytes and villous atrophy. Prior reports indicate variable histological features in the GI tract of GS patients. Duodenum of GS patients may show villous blunting [3]. Jansen et al. reported one case each of celiac disease and ulcerative colitis in their cohort of GS^2^. Apoptotic colopathy has also been described in GS patients [5]. Phlegmonous gastritis with causative organism as *Streptococcus oralis* was noted in one case [6]. Infections such as CMV, *Campylobacter*, *Giardia lamblia*, *Salmonella* species and bacterial overgrowth have been implicated as cause of diarrhea in GS patients [7]. On similar lines, case no. 1 manifested symptoms secondary to chronic norovirus infection.

As with histological features, similar variabilities exist in the immunologic and clinical landscape of GS patients. Some patients display no clinical abnormalities despite having abnormal immunological profiles while others may suffer from recurrent opportunistic infections with a relatively normal immunological profiles [8]. The onset of symptoms related to infections may precede, be concurrent, and even succeed the thymoma diagnosis [2, 9]. Our case cohort showed development of infectious complications years after thymectomy. Some GS patients can develop malignancies such as skin cancer, large granular T cell lymphoma, and thyroid cancers as well as recurrence of thymoma [2]. One of our patients developed an adenocarcinoma of the colon; however, we cannot establish a correlation owing to limited sample size.

Controversies exist if GS is its own separate entity or whether it is a subset of common variable immunodeficiency (CVID), resulting in underappreciation of GS. Several features confound the distinction between the two entities such as (1) later age of onset of immunodeficiency, (2) similar forms of immunodeficiency, and (3) associated clinical characteristics. Despite these, subtle differences exist. While presence of thymoma is a defining feature for GS patients, they tend to present without discernible family history, low/absent peripheral blood B lymphocytes, absence of lymphoid hyperplasia, and variable plasma cell infiltration in the lamina propria in some patients [3]. GS patients are more prone to encounter opportunistic infections due to additional defects in cell-mediated immunity. CVID patients are more likely to develop organomegalies such as hepatomegaly, splenomegaly, and lymphadenopathies [10]. The classic autoimmune association of thymomas is myasthenia gravis; however, GS is distinctive and is associated with peculiar set of autoimmune conditions, namely oral lichen planus, graft versus host disease-like colitis, and pure red cell aplasia [10]. CVID patients show a spectrum of changes such as active chronic inflammation, villous blunting, changes mimicking graft versus host disease, granulomatous inflammation, and nodular lymphoid hyperplasia, although the most appreciated association of CVID is lack of plasma cell infiltrate in the lamina propria in pathology specimens [11–13]. None of the reviewed slides in our cases showed presence of apoptotic enteritis/colitis, granulomas, or nodular lymphoid aggregates. The genetic alterations underlying GS are still unknown, while genetic causes are identified in 10% of cases of CVID [14].

Recognizing GS is important as they have a lower survival rate compared with CVID or X-linked agammaglobulinemia (70% at 5 years for GS versus almost 100% at 5 years for others) [15]. An appreciation of histomorphological findings in the GI tract may help in their differentiation from other immunological disorders, although differentiation from CVID needs a constellation of clinical findings. Paucity of plasma cells, though widely known to be associated with CVID, cannot be used as a defining feature in either CVID or GS. This histologic finding needs to be contextualized with clinical features, family history, and genetic findings, wherever available. Clarity of communication with the treating physician is vital since GS is an underappreciated form of immunodeficiency and could be mistaken for CVID clinically.

To conclude, we characterized histopathological details in two Good’s syndrome patients and shed light on their resemblance to CVID. Despite being discovered > 70 years ago, the immunological basis of GS remains incompletely understood, and we hope focused research will elucidate potential therapies and drug targets.
